# Economic Costs of Violence Against Women and Girls in Low- and Middle-Income Countries: A Pilot Study on Management’s Outlook

**DOI:** 10.1177/21650799221081262

**Published:** 2022-04-18

**Authors:** Mrinal Chadha, John Kennedy, Nata Duvvury

**Affiliations:** 1 National University of Ireland, Galway; 2 Ipsos MORI

**Keywords:** violence against women and girls, management, businesses, workplace violence, economic costs

## Abstract

**Background:**

In low and middle-income countries (LMICs), violence against women and girls (VAWG) is rampant, primarily due to patriarchy. However, there is little understanding of its ripple effect in the workplace in LMICs. While recent studies in LMICs have attempted to understand the effects of VAWG on productivity using data collected from colleagues, survivors, or perpetrators, there is limited research on the employers’ perspective of the impact of VAWG on productivity.

**Methods:**

A survey, developed by the investigators, based on previous research in Peru and Vietnam, was administered to 74 senior management executives in Ghana, Pakistan, and South Sudan. Based on female employees’ absenteeism, tardiness, and presenteeism, this study provides the management’s perspective on the invisible costs of VAWG.

**Findings:**

The results show that 25% and 36% of senior executives have witnessed intimate partner violence and non-partner sexual violence, respectively, against their female colleagues. One (32%) in three managers also acknowledged the impact of VAWG on productivity and day-to-day operations.

**Conclusions:**

This study provides evidence that there is a need for the development of employee assistance programs (EAPs) in LMICs. Due to significant increase in employees’ productivity in the absence of VAWG, investing in occupational health services needs to be viewed as an investment, not cost. As many international companies in developed countries do business in LMICs, their occupational health departments need to be more aware of VAWG. Occupational health practitioners can assist in the needs assessment for EAPs as well as provide appropriate referrals and counseling to impacted employees.

## Background

Globally, almost one third of women (30%) have experienced physical and/or sexual intimate partner violence (IPV) or non-partner sexual violence (NPSV) or both in their lifetime (World Health Organization [WHO], 2021). The literature is clear that there are physical and mental health impacts of violence in low- and middle-income countries (LMICs) (Asante et al., 2019; Elmusharaf et al., 2019; Ghaus et al., 2019). International policy, which is committed to women’s empowerment, has driven a steady increase in women’s participation in the paid workforce. It is thus important to understand whether IPV and NPSV impact businesses in terms of productivity. For businesses, the rate of absenteeism, tardiness, and work distraction (or presenteeism) among employees are seen as the three dimensions of productivity loss critical to the bottom line (Vara Horna, 2013). Equally, the impacts of IPV and NPSV at the workplace are important from a social reputation perspective as distressed employees can undermine client relationships, which represent the social capital of a business (Walker & Duvvury, 2016).

The U.S. literature recognized IPV-related work impacts, including unemployment, job instability and performance (Anderson et al., 2014; Crowne et al., 2011), missed workdays, tardiness, work disruption (Blodgett & Lanigan, 2018), and maintaining employment (Borchers et al., 2016). These work outcomes arising from IPV have also been documented in other developed countries, including Norway (Alsaker et al., 2016), Australia (McFerran, 2011), New Zealand (Rayner-Thomas, 2013), and Canada (Wathen et al., 2015). In contrast, research on work-related consequences of violence against women and girls (VAWG) in LMICs is scant (Chadha et al., 2020; Darko et al., 2015; Gupta et al., 2018). Occupational health providers based in country in LMICs and corporate occupational health leaders need to be aware of the dimensions of VAWG in the locations in which their company does business.

Ghana, Pakistan, and South Sudan are highly patriarchal societies, but at different levels of economic, social, and political stability (Asante et al., 2019; Elmusharaf et al., 2019; Ghaus et al., 2019).These countries, being LMICs, were selected as part of a larger study exploring the economic and social costs of VAWG in differing political contexts: stability (Ghana), fragility (Pakistan), and conflict (South Sudan). Across the three countries, Ghana and South Sudan have less restrictive norms on women’s mobility, with 63.6% and 70.7% women in labor force, respectively, compared with Pakistan (23.1%) (International Labour Organization [ILO], 2020). However, in all three countries, VAWG is normalized particularly around IPV (Asante et al., 2019; Elmusharaf et al., 2019; Ghaus et al., 2019).

In terms of labor protections of women workers, the labor laws in all three countries are grounded in the principle of no discrimination based on gender (Labour Act, 2003, 2017 Protection Against Harassment of Women at the Workplace Act [PAHWWA], 2010). In Ghana, section 175 of the 2003 Labor Act categorizes sexual harassment as a workplace offense (Labour Act, 2003). In Pakistan, the PAHWWA (2010) introduced the definition of harassment at the workplace and makes provisions to prevent it. In South Sudan, the Labour Act (2017) offers protection for local South Sudanese workers in terms of job security, where workers cannot be fired without reason, but there is no specific legislation on sexual harassment. In all three countries, labor laws do not specifically recognize IPV as a workplace issue.

The study has three goals. First, this study aims to understand the awareness of managers regarding their female employees’ experience of intimate partner violence (IPV) or nonpartner sexual violence (NPSV). Second, this study aims to understand the managers’ perceptions regarding the productivity impacts due to IPV and NPSV. Finally, this study aims to add to the limited literature on IPV from LMICs.

## Method

This is a pilot study of managers in businesses in three LMICs. Purposive quota sample was used within selected cities in each country. National researchers, guided by the Principal Investigator (N.D.), formulated a sampling strategy to cover businesses in the formal business sector. Research teams contacted a representative of each of the selected businesses in the sample, with 242 businesses refusing or unavailable to participate. Interviewers conducted a face-to-face standardized open-ended survey with the business representative. The business representatives generally comprised managers, chief executive officers, chief financial officers, and directors, among others (referred to as managers for simplicity hereinafter).

The survey collected information regarding managers’ understanding of violence, their knowledge of violence, and the impacts of violence within the workplace. The survey questions were guided by previous research in Peru and Vietnam (Duvvury et al., 2012; Vara Horna, 2013). The questionnaire incorporated the following: general statements about the company (such as what it is like working there, staff turnover, and whether the company would grant leave at short notice); reasons for employees’ absence during the past 4 weeks; measures the company has in place to cover for routine and unplanned absence from work; awareness and nature of employee experiences of violence/harassment in the past 12 months; consequences of the experiences on work attendance and productivity; business policy frameworks (like company policies or program to support female employees, training of supervisors and managers to assist female employees, support services offered); the overall cost of violence to the company within the last 12 months; what, if anything, further the company could do to ensure female employees have a good quality of life. The managers’ responses were collected predominantly using preset answer options. For instance, to enquire about the information on managers’ tenure, the preset options were the following: less than 12 months, over 1 and up to 2 years, over 2 and up to 5 years, over 5 and up to 10 years, more than 10 years, and don’t know. For information on witnessing or awareness about female employees’ experience of IPV and/or NPSV, the options were yes, no, don’t know, and prefer not to say. The expected productivity impacts due to IPV and/or NPSV were also laid out as shown in Table 2, and the managers had to choose from options of yes, no, don’t know, and prefer not to say.

The following definitions of violence have been used to gather information from the managers:

**IPV:** IPV is defined, either singly or in combination, as “being slapped, hit, kicked, beaten, choked, burned, or otherwise physically hurt; being threatened with such incidents; being threatened that others will experience such incidents; being prevented from leaving their home; attending work or other functions; being forced or coerced against their will to engage in sexual activity; being verbally insulted or humiliated; and having their money taken against their will” by an intimate partner of the woman.
**NPSV/Harassment (NPSV/H):** NPSV/H includes “being touched inappropriately; forced or coerced against their will to engage in sexual activity (excluding physical interaction); being called sexually derogatory names, or experiencing repeated unwanted sexual advances or comments” by a non-partner of the woman.
**Non-Partner Sexual Assault:** It is defined as unwanted physical touch, sexual in nature, with or without penetration by a non-partner of the woman.

Data were analyzed using Stata. Most of the collected data were descriptive in nature with no domain, particularly measured as scales, for which reliability and validity need to be established. Nonetheless, the domain that measured the working quality for the company was established as a scale. Cronbach’s alpha provided reliability of .7, which shows an acceptable level of internal consistency between the items. Correlation coefficients were used to establish convergent validity between different items, which suggested a moderately positive correlation between different items.

To ensure confidentiality, no employee was surveyed in the same business about their experience regarding violence as the managers. No incentives were provided to any businesses that participated in the research. In Ghana and South Sudan, local country teams advised that manager surveys would not be required in languages other than English. It was advised that those who worked in the formal business sector in these countries would be able to understand questions in English. In Pakistan, the surveys were conducted in Urdu/Sindhi. For translating surveys in Pakistan, a local fieldwork agency conducted translation, which was further proofread by a translation partner, cApStAn.

The NUI Galway Research Ethics Committee granted the approval for the study. The research protocol was approved in countries by the relevant ethics committees: University of Ghana Ethics Committee (Ghana), the National Bioethics Committee (Pakistan), and the National Bureau of Statistics (South Sudan).

This study has also attempted to understand the bivariate association of reported IPV and NPSV/H impacts considering the country, gender, and size of the firm (small, medium, and large). Pearson’s chi-square test and Cramer’s *V* have been used to establish the bivariate associations and their strength.

## Results

### Descriptive Statistics

The survey response rate was 23.4%. A total of 74 managers, employing female employees, were interviewed face-to-face: 25 in Ghana, 27 in South Sudan, and 22 in Pakistan.

As shown in Table 1, most managers have been working with their respective companies for a long time, with <7% working for <2 years. The majority of the managers surveyed were male (86%).

**Table 1.** Gender, Length of Employment, and Gender of Managers (*n* = 74).

Characteristics of Employees and Managers	Ghana	South Sudan	Pakistan	Total
	(*n* = 25)	(*n* = 27)	(*n* = 22)	(*n* = 74)
	*n* (%)	*n* (%)	*n* (%)	*n* (%)
Gender (%)				
Male	25 (68)	27 (57)	22 (78)	74 (67)
Female	25 (32)	27 (43)	22 (22)	74 (33)
Length of employment (%)				
Less than 2 years	0 (0)	0 (0)	5 (23)	5 (7)
Over 2 and up to 5 years	11 (44)	6 (22)	7 (32)	24 (32)
Over 5 and up to 10 years	8 (32)	21 (78)	5 (23)	34 (46)
More than 10 years	6 (24)	0 (0)	5 (23)	11 (15)
Gender of employee’s manager (%)				
Male	19 (76)	24 (89)	21 (95)	64 (86)
Female	6 (24)	3 (11)	1 (5)	10 (14)

*Source*. Authors’ own.

Table 2 shows that one (25%) in four managers knew or suspected that their female employees were suffering from any form of violence from their husband, live-in partner, boyfriend, or fiancé. One fifth (20%) of managers were told by female work colleagues that they have been harassed or threatened by their spouse or partner while at work; the threat might have been in person, by phone, email, or text message. One (17%) in six managers witnessed their female employees being verbally abused, insulted, or humiliated by their partner while at work. In terms of physical abuse, 6.2% also witnessed female employees being physically hit or beaten by their partners while at work.

**Table 2.** Managers Reporting Productivity Impacts Due to IPV and NPSV Experienced by Women Employees (*n* = 27).

Productivity impacts	% of managers aware of IPV against female employees reporting impacts^a^	% of managers reporting impacts of those who witnessed NPSV/H
	*n* (%)	*n* (%)
Arrived late by an hour or more	8 (57)	2 (15)
Left early by an hour or more	5 (33)	6 (43)
Missed appointments at work	6 (43)	6 (46)
Missed work for one or more days	9 (60)	6 (43)
Not as productive as normal	9 (60)	14 (82)
Had difficulties dealing with customers or clients	8 (53)	11 (65)
Had difficulties working with colleagues	4 (33)	14 (78)
A manager raised concerns about their work	6 (43)	6 (43)

a The base for percentages is not same as some managers mentioned “prefer not to say” and were thus excluded for that particular impact.

Nearly four (36%) in 10 managers reported knowing or suspecting female employees experiencing NPSV/H in the past 12 months. About one third (35%) reported being approached by females employees to request advice or support regarding NPSV/H in the past 12 months. Roughly 4% of managers report survivors disclosed experiencing NPSV/H in their homes. Almost 13% of managers report survivors experiencing NPSV/H on the way to and from work, and a very high percentage of managers (33%) reported survivors experiencing it at work.

Managers also reported survivors of both IPV and NPSV/H experiencing tardiness (getting late to work), absenteeism (missing work), and presenteeism (being less productive at work). As presented in Table 2, 57% of managers who were aware of their female employees experiencing IPV reported survivors arriving late by an hour or more. Six (60%) in 10 managers reported survivors missing work for one or more days as well as being less productive. Similarly, 53% of managers reported survivors had difficulties dealing with customers or clients, and almost 43% reported that managers raised concerns about the work of IPV survivors.

Managers also reported some form of productivity impacts due to NPSV/H in women survivors, with almost 43% of managers seeing NPSV/H survivors leaving work by an hour or more and missing work for one or more days. Presenteeism was also evident among NPSV/H survivors, with 82% of managers reporting survivors being less productive and almost 78% reporting NPSV/H survivors having difficulties working with colleagues.

The productivity impacts of both IPV survivors and NPSV/H survivors were in sync with the previous research, which also found survivors of IPV in Ghana, Pakistan, and South Sudan reporting significant productivity loss (Asante et al., 2019; Elmusharaf et al., 2019; Ghaus et al., 2019).

### Non-Partner Sexual Assault

One (25%) in four managers reported witnessing female employees being sexually assaulted at work (e.g., unwanted physical touching of a sexual nature, with or without penetration) within the past 12 months. In terms of the type of individuals by which women employees experienced non-partner sexual assaults at work, 72% of the managers who witnessed assaults (*N* = 10) reported a stranger or a work colleague to be the perpetrator, and 21% reported someone else known to the women to be the perpetrator.

### Overall Impact of Violence

The overall impact of IPV or NPSV/H is borne primarily by survivors or their colleagues. Thus, 18% of the managers, whose female employees experienced IPV or NPSV/H, reported women survivors leaving the company permanently as a result of violence. Disturbingly, 8% of the managers whose female employees experienced IPV or NPSV reported female employees being sacked from the company as a result of violence. The company’s response to female employees missing work resulted in the majority of the burden falling on existing colleagues, with only 4% of companies recruiting additional staff and 7% recruiting additional permanent staff. Similar to the impacts of IPV or NPSV/H, the majority of the impact of sexual assaults was borne by the survivors or their colleagues.

### Bivariate Association

The results of the bivariate association are shown in Table 3. There was a statistically significant association between country and productivity impacts due to IPV. Cramer’s *V* values ≥0.8 show a strong effect. This implies that productivity impacts do differ in different countries. A statistically significant association was also found between the gender of managers and arriving late by 1 hour, as well as missing work by more than 1 day. In other words, there is a statistically significant difference in male and female managers reporting female survivors arriving late by 1 hour and missing work by more than 1 day. In the case of NPSV/H, leaving early by 1 hour or more was associated with country, and missed appointments at work were associated with the gender of managers. In other words, there is a statistically significant difference across countries in survivors leaving early by 1 hour or more, and male and female managers reporting survivors missing appointments at work. No statistically significant association was found between the size of the firm and IPV or NPSV/H.

**Table 3.** Bivariate Associations (*n* = 27).

Productivity impacts	Intimate partner violence			Non-partner sexual violence/harassment		
	Countries	Managers’ gender	Size of the firm	Countries	Managers’ gender	Size of the firm
Arrived late by an hour or more	8.2 (0.8)**	3.1 (0.5)*	3.8 (0.5)	0.7 (0.2)	0.4 (0.5)	3.8 (0.5)
Left early by an hour or more	8.6 (0.8)**	1.2 (0.3)	1.6 (0.3)	2.9 (0.5)*	0.05 (0.1)	0.1 (0.1)
Missed appointments at work	14 (1)***	1.8 (0.4)	3.3 (0.5)	0.3 (0.1)	2.8 (0.5)*	4.4 (0.6)
Missed work for one or more days	9 (0.8)**	3.5 (0.5)*	0.4 (0.2)	0.9 (0.3)	1.8 (0.4)	2.1 (0.4)
Not as productive as normal	11.4 (0.9)***	0.1 (0.1)	4.4 (0.5)	0.8 (0.2)	0.8 (0.2)	3.2 (0.4)
Had difficulties dealing with customers or clients	11.6 (0.9)***	2.6 (0.4)	2.9 (0.4)	1.6 (0.3)	1.9 (0.3)	1.4 (0.3)
Had difficulties working with colleagues	12 (1)***	1.2 (0.3)	1.9 (0.4)	0.3 (0.1)	0.6 (0.2)	1 (0.2)
A manager raised concerns about their work	2.7 (0.4)	1.8 (0.4)	3.3 (0.5)	0.9 (0.3)	0.05 (0.1)	1.2 (0.3)

*Source*. Authors’ own.

*Note*. The figures in tables represent Pearson’s chi-square test with Cramer’s *V* in parentheses;.

As countries, managers’ gender, and size of the firm as well as productivity impacts are nominal variables, their association is established using Pearson’s chi-square test, with Cramer’s *V* showing the strength of association. For example, managers reporting employees “arriving late by an hour” is associated with countries (chi-square = 8.2, *p* < .05). This implies a statistically significant difference is observed across countries about this productivity impact.

*** *p* < .01. ** *p* < .05. * *p* < .1.

### Prevention and Response to Gender-Based Violence

Managers were also asked about special measures to assist female employees in general, as well as those experiencing IPV or NPSV/H. The majority of the companies had no specific assistance schemes for female employees, with about 19% providing transport or accommodation for late-night work. For violence survivors, 25% of managers report medical assistance, and loans or financial assistance was offered. When asked about what the company can do for enhancing the quality of lives of female employees due to gender-based violence, the majority of managers felt that company already does a lot, and nothing else is needed (47%). The second most common theme that emerged from managers’ responses was about providing a better environment in terms of respect and support for female employees (20%). This was followed by approximately 14% of managers suggesting either a need to develop a new gender-based policy or revising the existing ones. The least common themes were providing free services to survivors, such as health care and transport, or providing some form of economic benefit, such as higher salaries/allowances, life insurance, and health insurance.

## Discussion

This study addresses some critical gaps in the literature by uncovering managers’ perspectives on productivity impacts due to VAWG, as well as the concrete actions taken by the managers in the context of LMCs. While the ILO190 convention makes addressing IPV and sexual violence at the workplace an obligation (International Labour Organization [ILO], 2019), this study points out that businesses also have an economic incentive in addressing them. Survivors of both IPV and NPSV/H report being late to work, absent from work, as well as being less productive. This in turn affects the output of businesses. As shown in previous research, the benefits of reducing VAWG generally outweigh the costs of interventions (Chadha et al., 2020).

Equally important is to reduce and prevent VAWG as a part of the company response. In the context of LMICs, a recent study by Al-Mamun et al. (2018) validated the intervention “HERrespect” in reducing both IPV and workplace violence against female garment workers in Bangladesh. Similar interventions that can be adapted to LMC contexts need to be undertaken. An unaddressed question in the literature is regarding workplace policies to hold perpetrators accountable. The high level of sexual violence and harassment reported in the study highlights the urgency of developing robust mechanisms at the workplace. Specific leave policies and flexible working arrangements are required to provide support for the recovery of survivors. Legislation mandating leave for domestic violence has been adopted in several countries, including New Zealand, Australia, and Canada.

### Implications for Occupational Health Practice

This study has several implications for occupational health practice, especially given the low availability of occupational health services in these countries. First, this study established the strong need for the development of employee assistance programs generally, which are currently very fragmented in these countries. These programs in the Global North do provide some evidence on supporting survivors of IPV (Lindquist et al., 2010; Pollack et al., 2010), but they are far less than sufficient. Second, given the high prevalence of IPV in these countries, occupational health practitioners need to incorporate into their clinical practice the assessment of possible long-term ill health, both physical and mental, as a consequence of IPV. Third, as many international companies in developed countries do business in LMCs, their occupational health departments need to be more aware of VAWG, which is prevalent in many LMCs. Finally, this study points to the significant increase in employees’ productivity in the absence of VAWG. Hence, by investing in occupational health services, which businesses see as an additional cost, the resultant gain in the form of increased productivity has the potential to far outweigh the cost of providing these services.

#### Limitations and future research

There are primarily two limitations to this study. First, it is based on a small sample size of 74 managers; therefore, the results cannot be generalized for the three countries, that is, Ghana, Pakistan, and South Sudan. Future research can replicate the study by taking a large sample size and comparing the results. Second, to ensure the confidentiality of survivors, this study did not include the voice of victims from the same company as those of managers. Thus, we were not able to elaborate on the issue of power and control in the workplace in any depth. In the future, a study can be designed that would include the voice of victims and their perceptions of IPV and NSPV/H impact, as well as their ability to report violence to management, and thus looking at the differences with those of the managers. Future research can also explore workplace policies that facilitate holding perpetrators accountable.
